# Prognostic utility of interim ^18^F-FDG PET/CT after two cycles of ABVD in response assessment in Hodgkin’s lymphoma patients: single-center preliminary experience

**DOI:** 10.22038/aojnmb.2025.89081.1644

**Published:** 2026

**Authors:** Nguyen Quang Toan, Pham Van Thai, Le Thanh Dung, Mai Hong Son, Pham Lam Son, Do Huyen Nga, Le Van Quang, Le Ngoc Ha

**Affiliations:** 1 Department of Nuclear Medicine, National Cancer Hospital and Hanoi Medical University, Hanoi, Vietnam; 2 Department of Radiology, Viet Duc Hospital, Hanoi, Vietnam; 3 Department of Nuclear Medicine, 108 Military Hospital, Hanoi, Vietnam; 4 Department of Oncology, National Cancer Hospital and Hanoi Medical University, Hanoi, Vietnam

**Keywords:** Keywords:Interim ^18^F-FDG PET/CT, Early response treatment Progression-Free Survival Hodgkin’s Lymphoma

## Abstract

**Objective(s):**

This study evaluates the utility of interim ^18^F-FDG PET/CT (iPET)-guided therapy in a Southeast Asian population, addressing gaps in region-specific data. Key outcomes included treatment response rates and progression-free survival (PFS) stratified by iPET results (Deauville score (DS) 1-3 vs. 4-5) across all clinical risk groups (including early-stage favorable/unfavorable and advanced-stage based on the International Prognostic Score (IPS)). Findings will inform optimal risk-adapted strategies in resource-aware settings.

**Methods:**

A prospective study was conducted of 100 patients with Hodgkin Lymphoma (HL) at the Vietnam National Cancer Hospital from March 2020 to March 2024. All patients underwent baseline clinical assessment and imaging (CT and/or PET/CT), followed by two cycles of ABVD chemotherapy. IPET was performed for early response assessment using Deauville scores (DS), with subsequent treatment adjusted according to NCCN guidelines.

**Results:**

A total of 100 patients with classical Hodgkin lymphoma (mean age: 32±13.8 years; range 9-73) were analyzed. Bulky disease and extranodal involvement were observed in 10.0% and 15.0% of cases, respectively. Early-stage disease (stage I-II) was present in 72.0%, and advanced-stage (stage III-IV) in 28.0%. After two cycles of ABVD, 78.0% of patients had a negative iPET result (DS 1-3), of whom 88.5% were DS 1, while 22.0% had a positive iPET result (DS 4-5), predominantly DS 4 (72.7%). In early-stage disease, the 3-year progression-free survival (PFS) was significantly higher in the favorable group than in the unfavorable group (95.7% vs. 81.2%, p=0.03). In advanced-stage disease, low-risk (IPS 0–3) patients achieved a 3-year PFS of 88.2%, whereas high-risk (IPS 4-7) patients had a markedly lower PFS of 42.9% (p<0.001). Overall, patients with negative iPET had substantially better 3-year PFS than those with positive iPET (93.6% vs. 40.9%, p<0.0001). The predictive performance of iPET for treatment outcomes showed a sensitivity of 72.3%, specificity 89.0%, PPV 59.0%, NPV 93.6%, and overall accuracy 86.0% (95% CI 0.78-0.91). Diagnostic accuracy remained high across subgroups, ranging from 84.0% in early-stage disease to 89.5% in advanced-stage, and was highest in favorable early-stage (90.8%) and low-risk advanced-stage (93.7%) patients. In multivariate analysis, iPET was identified as an independent predictor of PFS (p<0.05).

**Conclusion:**

In a real-world Vietnamese cohort with Hodgkin lymphoma, interim PET/CT guided by Deauville scoring after two cycles of ABVD chemotherapy showed strong predictive value for treatment response. The results advocate for broader integration of NCCN-consistent risk-adapted strategies in Southeast Asia.

## Introduction

 Hodgkin lymphoma (HL) has an annual incidence of approximately 0.98 per 100,000 people worldwide (1, 2). It is most common in young adults (20–30 years), with a smaller peak after age 65 (3). HL is highly sensitive to standard chemotherapy, radiation therapy, or combined-modality therapy, with long-term cure rates expected to be more than 80% in patients receiving the standard treatment (4). The primary objective is to mitigate treatment-related adverse effects while maintaining therapeutic efficacy. ABVD chemotherapy, the standard regimen for all HL stages, offers a favorable balance between clinical effectiveness and reduced toxicity compared to alternative therapies. 

 FDG PET/CT has been widely used for staging and assessing treatment response in HL (5-7). iPET assessment allows for early response evaluation during treatment and has been demonstrated to predict therapy outcome at an earlier stage of treatment. It also serves as an early predictor of response, allowing risk-adapted treatment strategies. The Deauville five-point score (DS) has been utilized as a standard criterion in early response assessment after two cycles of ABVD chemotherapy in HL patients (6-9). iPET is usually performed after a few initial cycles of chemotherapy and is a better prognostic tool than the International Prognostic Score (IPS), followed by the treatment guideline by the US National Comprehensive Cancer Network (NCCN) and the European Society for Medical Oncology (ESMO) (8, 9). In addition, patients with iPET (–) in early-stage HL were given 4–6 cycles of ABVD chemotherapy, while those with advanced-stage disease were treated with 6 to 8 cycles of ABVD. Megavoltage radiotherapy was delivered to bulky disease sites or residual lymphoma lesions following chemotherapy. For patients with iPET(+) after two cycles of ABVD, escalation treatments, including BEACOPP chemotherapy and/or autologous stem cell transplantation (ASCT), were administered based on the response status and decided by a multidisciplinary team. No therapy change was made based on iPET unless disease progression was documented by CT or PET/CT, with biopsy when feasible (10, 11).

 Epidemiological and biological differences exist between Asian and Western HL populations particularly the higher prevalence of EBV-positive classical HL in Asia (up to 80–100% in children and approximately 65% in adults). These differences may contribute to distinct tumor microenvironments and variable responses to chemotherapy and immuno-therapy (2, 12).

 Despite the widespread adoption of NCCN and ESMO guidelines, these frameworks are primarily based on Western clinical trials and may not fully account for regional differences in disease biology, healthcare infrastructure, or patient demographics. In Southeast Asia, real-world evidence validating such guidelines remains limited.

 To address this gap, our study evaluated the prognostic value of interim PET/CT using the DS after two cycles of ABVD chemotherapy in HL patients. We focused on its predictive performance for treatment response and PFS, and assessed the applicability of NCCN-recommended iPET-adapted strategies in a Southeast Asian cohort (8).

## Methods

***Patient*** ***s***

 This was a single-center prospective study conducted at the National Cancer Hospital in Hanoi, Vietnam. Eligible patients had: (1) newly diagnosed, histologically confirmed classical Hodgkin lymphoma (stage I–IV); (2) treatment with a response-adapted strategy per NCCN guidelines (e.g., ABVD ± radiotherapy for iPET(–) patients or escalation to BEACOPP for iPET(+) patients); and (3) available imaging for both baseline staging (CT or PET/CT) and response assessment (interim PET after two ABVD cycles and/or end-of-treatment FDG PET/CT).

 Notably, FDG PET/CT was not uniformly employed for initial staging during the early phase of the study, as its use depended on equipment availability and clinical discretion. Consequently, only about one-third of patients underwent PET/CT-based staging. Patients with a prior history of lymphoma or other malignancies (except basal cell carcinoma) were excluded.

 The disease stage was assigned based on the Ann Arbor staging system (15) . Early-stage disease (stage I or II) is typically confined to a single lymph node area or a limited number of lymph node areas, and potentially an organ of the lymphatic system or an extranodal site. An advanced stage (stage III or IV) indicates the cancer has spread beyond the lymph nodes, potentially involving organs or bone marrow. 

 Bulky mass was defined according to NCCN guidelines as the presence of a mass larger than 10 cm in diameter (8). The WHO 2008 classification schema recognizes two histological types of HL: the nodular lymphocyte predominant and the “classic” HL. The latter encompasses four entities: nodular sclerosis, mixed cellularity, lymphocyte depletion, and lymphocyte-rich (15).

 Early-stage patients were categorized as favorable and unfavorable based on the presence of ≥1 of the following factors: extranodal disease, bulky mediastinal mass >10 cm, erythrocyte sedimentation rate>50, ≥3 disease sites, and age ≥45 years. For advanced disease, the International Prognostic Score (IPS) was applied. Patients were categorized into low-risk (IPS 0–3) and high-risk (IPS 4–7) groups (16). Clinical, demographic, and risk factors were extracted from medical records. Laboratory parameters were dichotomized using NCCN-defined thresholds: hypo-albuminemia (<4.0 g/dL), anemia (hemoglobin <10.5 g/dL), leukocytosis (WBC ≥15,000/mm³), and lymphocytopenia (lymphocyte count <600/mm³ and/or <8% of WBC). 

 Patients subsequently underwent ABVD chemotherapy per NCCN guidelines (8). iPET was performed after two treatment cycles to assess early therapeutic response using DS and guide subsequent regimen selection. The Deauville score has been proposed as a rapid qualitative method to evaluate iPET through visual comparison between the uptake within residual lymphoma tissue and the reference regions, the mediastinum and live (17).

 Patients were categorized into two follow-up groups: iPET(–) (DS 1 to 3) and iPET(+) (DS 4 or 5). PFS of patients was also monitored during and after treatment. Patients’ clinical information was collected from the Electronic Medical Record (EMR) and PET/CT (SUV_max_) for analysis. In addition, treatment response was assessed using the Lugano response criteria (11).

**Figure 1:**
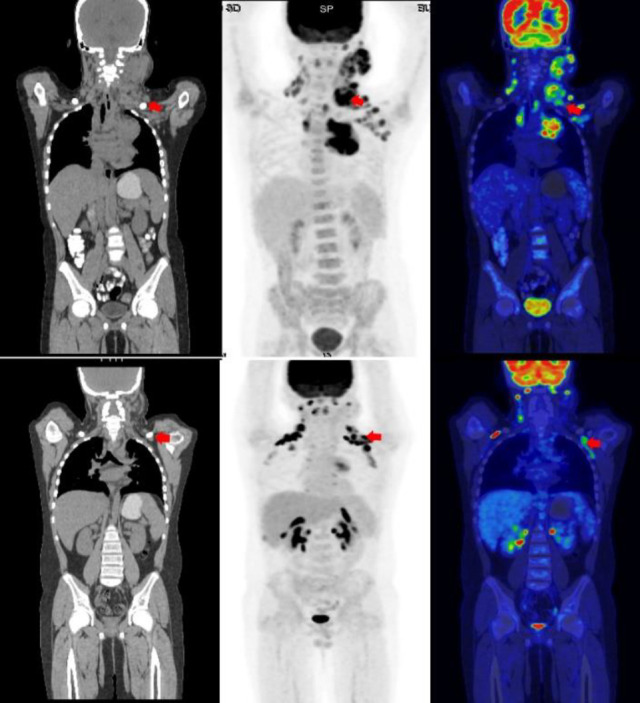
A 13-year-old male patient was diagnosed with classical Hodgkin lymphoma (cHL), mixed cellularity type, stage IIIA. Baseline PET/CT demonstrated FDG-avid lymphadenopathy in cervical, mediastinal, and axillary regions (SUV_max_: 23.4) (**A**-**C**, **red arrow**). After receiving 2 cycles of ABVD chemotherapy, interim PET/CT (iPET) showed significant reduction in lesion size but with residual metabolic activity exceeding liver uptake (Deauville score 4, partial metabolic response [PMR]), consistent with iPET-positivity (**D**-**F**, **red arrow**). Treatment was subsequently intensified to 4 cycles of BEACOPP, achieving complete metabolic response (CMR). The patient has maintained sustained remission without evidence of disease recurrence during follow-up

**Figure 2:**
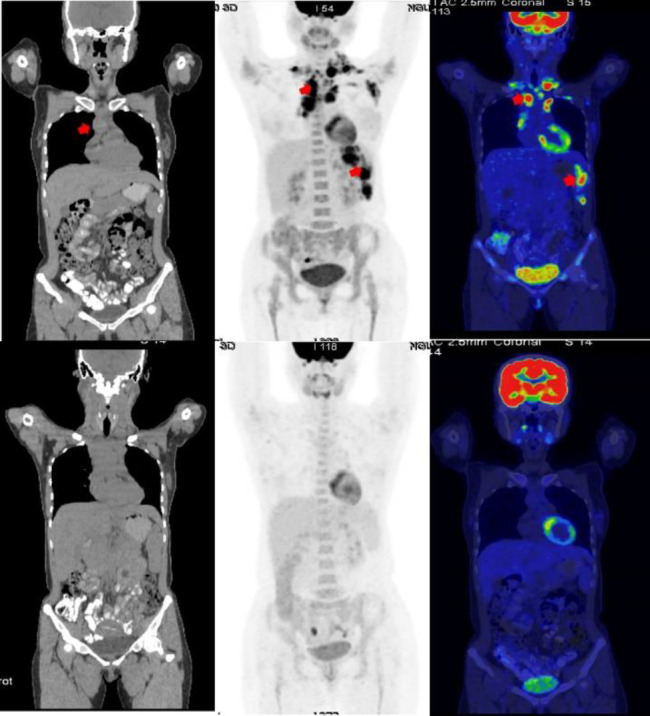
A 34-year-old female with stage IIIS mixed-cellularity classical Hodgkin lymphoma (cHL) presented with FDG-avid cervical/mediastinal lymph nodes (SUV_max_: 12.1) and splenic involvement (SUV_max_: 17.2) on baseline PET/CT (**A**-**C**, **red arrow**). After 2 cycles of ABVD, interim PET/CT revealed complete metabolic resolution (Deauville score 1), confirming complete metabolic response (CMR) (**D**-**F**). Treatment continued with 4 cycles of ABVD, achieving CMR with no recurrence on follow-up

 PFS was measured from the initiation of ABVD chemotherapy until the date of progression/ relapse based on CT or FDG-PET/CT. Persistent disease in a post-chemotherapy residual mass, based on biopsy or abnormal end of therapy FDG-PET/CT, with other clinical evidence of residual disease, was also considered. Death from any cause or last follow-up was recorded. Failure patterns, including disease progression, relapse, and death from any cause, were evaluated. These outcomes, along with the last follow-up data, were used to calculate the sensitivity (Se), specificity (Sp), negative predictive value (NPV), and positive predictive value (PPV) of iPET scans for predicting treatment outcome in both the iPET(–) and iPET(+) groups(Figures 1, 2). 

***Image acquisition and analysis***

 All patients fasted for more than 4-6 hours before undergoing FDG-PET/CT, ensuring a blood glucose level of less than 180 mg/dL. Images from the mid skull to the upper thigh were taken approximately 60 minutes after intravenous administration of 370 MBq F-18 FDG. PET/CT Discovery IQ with PET: 5 rings (GE Healthcare, Milwaukee, WI, USA) by the European Association of Nuclear Medicine (EANM) guidelines, version 2.0 (PCT 251700004PT-EARL standard) were used (18). 

 PET images were acquired in 3D mode position from the skull base to mid-thigh (Flow motion technology; table speed 1 mm/second equal to 3 min/bed). All PET images were reconstructed using an iterative algorithm and attenuation correction with low-dose CT images. Low-dose non-contrast CT parameters were: 120 kVp, modulated mAs, 3.75-mm slice thickness, 0.5-s rotation. ^18^F FDG PET/CT images were displayed in the trans-axial, sagittal, and coronal planes using the PET OncoViewer, GE workstation (version 4.7, GE Healthcare).

 All PET/CT scans were interpreted independently by two experienced nuclear medicine physicians, each blinded to the clinical and histopathological data. Both visual (Deauville

score) and semiquantitative (SUV_max_) assessments were performed. Interobserver agreement was assessed by calculating Cohen’s kappa statistic for Deauville scoring. In cases of discrepancy, the final interpretation was established by consensus in a joint session. No scan was excluded due to disagreement. For quantitative analysis, SUV_max_ was measured within the same lesion on both readers' assessments using a standardized protocol on a dedicated workstation. The lesion with the highest FDG uptake was selected for analysis.

***Statistical methods***

 Descriptive analysis and data frequencies were estimated. Data were analyzed using SPSS version 20.0 (SPSS Inc.) for statistical evaluation. A paired t-test was used to compare SUV_max_ and follow-up SUV_max_ values. Chi-square test or Fisher’s exact test was performed to identify clinical factors associated with disease progression. In addition, Kaplan-Meier analysis was performed to estimate PFS, and the log-rank test was used to identify prognostic factors. For PET/CT parameters (continuous variables), median values were used to classify patients into two groups. A Cox-regression test was performed to identify independent prognostic factors for PFS.

## Results

 Between June 2020 and June 2024, 100 patients with classical Hodgkin lymphoma (cHL) were enrolled. The mean age was 34±14 years, with a slight female predominance (female-to-male ratio 1.2:1). Nodular sclerosis was the most common histological subtype (52%) (Table 1).

**Table 1:** General characteristics of the cHL patients

**Patient’s characteristics**		**N (%)**
**Age**	Median (y, range)	34 (9-73)
	45	80 (80%)
	>45	20 (20%)
**Sex**	Females	45 (45%)
	Males	55 (55%)
** Symptoms B**		25(%25)
**Lymph nodal sites**	Supradiaphragmatic	63 (70%)
	Infradiaphragmatic	3 (3.3%)
	Both	24 (26.7%)
**Number of lymph node sites**	< 3 sites	55 (55%)
	≥ 3 sites	35 (35%)
	No lymph node	10 (10%)
**Histological type**	Nodular sclerosis	52 (52%)
	Mix cellularity	39 (39%)
	Lymphocyte depletion	9 (9%)
** Albumin <4.0 g/dl**		28 (28%)
** White blood cell>15,000/mm3**		13 (13%)
** Lymphocytes <600/mm3**		3 (3%)
** Hemoglobin <10.5 g/dL**		10 (10%)

***Baseline imaging and treatment characteristics***

 Initial staging was performed using FDG-PET/CT in 34% and CT in 66% of patients. Early-stage disease accounted for 72% of cases (57 favorable and 15 unfavorable), while 28% were advanced stage (21 low-risk and 7 high-risk by IPS).

 Treatment strategies followed NCCN guidelines, with early-stage patients receiving 2–6 cycles of ABVD ± radiotherapy and advanced-stage patients receiving ABVD or escalated BEACOPP based on interim PET (iPET) findings. Radiotherapy was delivered to 10 patients, primarily for bulky or residual disease (Table 2).

**Table 2:** Staging outcomes based on the baseline imaging used for the initial stage, prognostic factors, and risk stratification of cHL patients

**Parameters**			**N (%)**
**Total**			100 (100%)
**Stage**		I	16 (16.0%)
		II	56 (56.0%)
		III	17 (17.0%)
		IV	11 (11.0%)
Extranodal involvement			15 (15%)
Splenic involvement			12 (12.0%)
Bone and bone marrow involvement			5 (5.0%)
Mediastinum involvement			26 (26.0%)
Bulky site involvement			10 (10.0%)
Othersa			16 (16.0%)
**Early Stage (I-II)b**	Favorable		41 (56.9%)
	Unfavorable		31 (43.1%)
**Advanced Stage (III-IV)c**	Low risk (IPS 0-2)		17 (60.7%)
	High risk (IPS 3-7)		11 (39.3%)

CHL: Classical Hodgkin Lymphoma.^a^Lung**-**7, chest wall **- **3, liver - 2, adrenal gland** -**1.^ b^Categorized into favorable and unfavorable based on the presence of ≥1 of the following factors: extranodal disease, bulky mediastinal mass > 10 cm, erythrocyte sedimentation rate>50, ≥ 3 disease sites, and age ≥ 45 years. ^c^Low risk and high risk base on IPS

***iPET Response and Treatment Outcomes***

**Figure 3:**
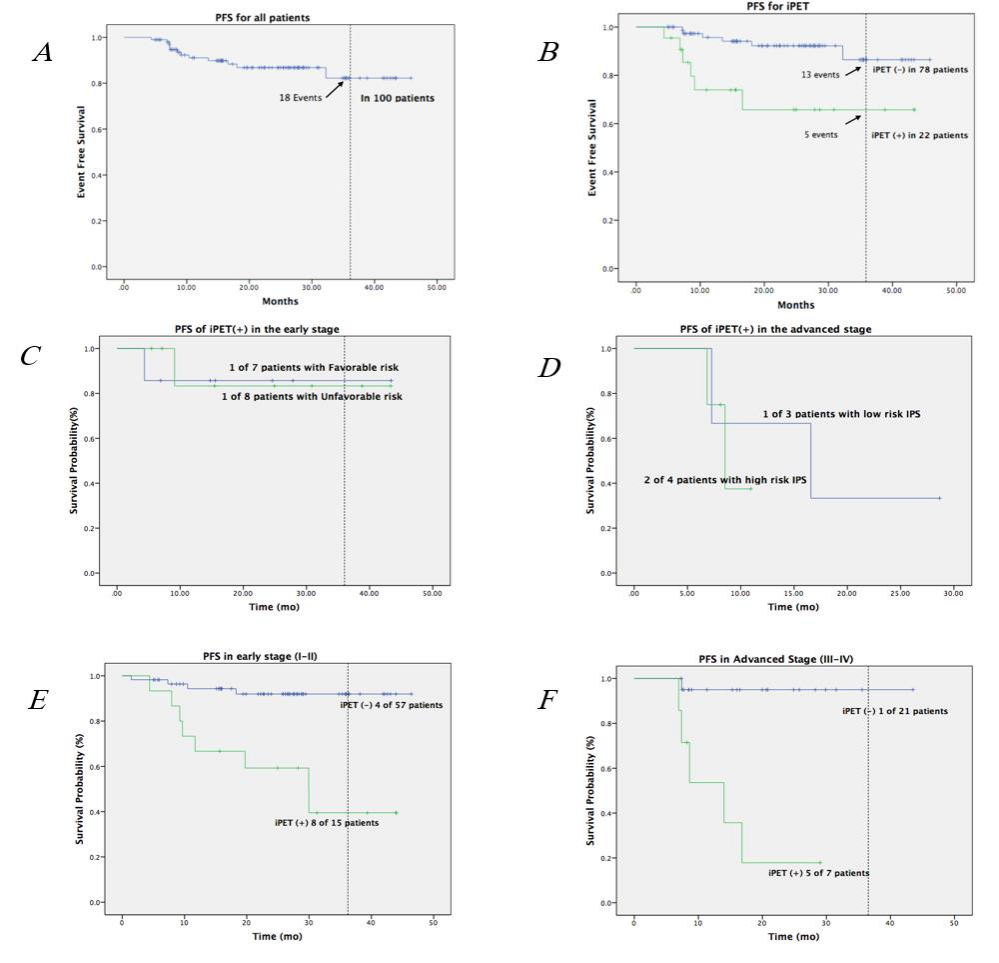
Kaplan–Meier curves illustrating progression-free survival (PFS) are shown for: (**A**) all patients with cHL; (**B**) patients stratified by iPET status; (**C**) favorable vs. unfavorable risk groups in early-stage disease; (**D**) low- vs. high-risk IPS groups among iPET-positive patients; and (**E**, **F**) early-stage vs. advanced-stage disease

 Overall, 82 of 100 patients (82%) achieved complete remission, while 18 (18%) experienced treatment failure (9 progression, 9 relapse). The 3-year PFS for the entire cohort was 82%. Patients with negative iPET had significantly superior 3-year PFS compared with iPET-positive patients (93.6% vs 40.9%, p<0.0001) (Figure 3).

 Among early-stage cases, PFS was 93.0% for iPET(–) versus 53.3% for iPET(+); in advanced-stage disease, PFS was 80.0% for iPET(–) versus 33.3% for iPET(+) (Table 3). Subgroup analysis confirmed consistently poor outcomes among iPET(+) patients regardless of IPS category.

**Table 3:** Treatment outcomes according to stage, risk group, and interim PET/CT results

**Stage and Prognostic Factor**	**iPET Result**	**Total N**	**CR**	**PRO**	**REL**	**N** **of Events** **(PRO + REL)**	**Event-Free (CR)**	**Event-Free %**	**3-year ** **PFS (%)** **[95% CI]**
Early Stage – Favorable/Unfavorable	iPET (–)	57	53	2	3	5	53	92.9%	Favorable 95.7%; Unfavorable 81.2%; *P* = 0.03
	iPET(+)	15	7	7	1	8	7	46.7%	
Subtotal		72	60	9	4	13	60	83.3%	
Advanced Stage – Low/High IPS	iPET (–)	21	20	0	1	1	20	95.2%	Low-risk 88.2%; High-risk 42.9%; *P* < 0.001
	iPET (+)	7	2	5	0	5	3	33.3%	
Subtotal		28	22	5	1	6	22	78.6%	
GRAND TOTAL		100	82	14	5	19	82	82.0%	

CR: Continued Complete Remission; PRO: Primary Refractory/Progression within 6 months after completion of therapy; REL – Late relapse after initial remission; iPET – interim PET/CT; 3-year PFS values were estimated using the Kaplan–Meier method; *P*-values derived from the log-rank test

***Diagnostic Performance of iPET***

 The diagnostic performance of interim PET/CT after two ABVD cycles is summarized in Table 4. Overall, iPET showed high predictive accuracy (86%**)** with a sensitivity of 72.3%, specificity 89.0%, positive predictive value 59.0%, and negative predictive value 93.0%. When stratified by clinical subgroup, accuracy remained excellent-90.8% in favorable early-stage**, **93.7% in low-risk advanced-stage, and slightly lower in unfavorable or high-risk groups-confirming the robustness and consistency of iPET across different disease categories.

**Table 4:** Predictive value of iPET after two cycles of ABVD in cHL patients with overall, stage, Favorable/Unfavorable, Low risk (IPS 0-2), High risk (IPS 3-7) group

**Group**	**Sensitivity**	**Specificity**	**PPV**	**NPV**	**Accuracy** **(95% CI)**
Overall	72.2% (0.49–0.87)	89% (0.80–0.94)	59% (0.38–0.77)	93% (0.86–0.97)	86.0% (0.78–0.91)
Early Stage (I-II)	66.7% (0.30–0.90)	88.6% (0.77–0.95)	53.3% (0.25–0.79)	92.6% (0.83–0.97)	84.0% (0.75–0.90)
Advanced Stage (III-IV)	83.3% (0.37–0.98)	90.9% (0.71-0.99)	71.4% (0.36–0.92)	90.0% (0.69–0.98)	89.5% (0.80–0.95)
Early Stage –Favorable	87.5% (0.47–0.99)	91.2% (0.76–0.98)	77.8% (0.4–0.96)	95.5% (0.81–0.99)	90.8% (0.82–0.96)
Early Stage –Unfavorable	50.0% (0.7–0.93)	95.7% (0.78–0.99)	75.0% (0.10–0.98)	91.3% (0.72–0.98)	87.3% (0.78–0.94)
Advanced Stage (IPS 0-2)	100% (0.47–1.0)	91.7% (0.61–0.99)	83.3% (0.36–0.99)	100.0% (0.73–1.0)	93.7% (0.85–0.98)
Advanced Stage (IPS 3-7)	100% (0.16–1.0)	88.9% (0.51–0.99)	50.0% (0.07–0.93)	100% (0.66–1.0)	92.5% (0.83–0.97)

***Treatment Failure and Salvage Therapy***

 Among 18 patients with treatment failure, 12 received second-line chemotherapy and 6 underwent autologous stem-cell trans-plantation (ASCT) either as part of salvage or frontline intensification strategies. In the iPET(–) group, only 5 of 78 (6.4%) relapsed or progressed, while 93.6% remained in continuous remission (Table 3).

***Prognostic Factors***

 The prognostic value of interim PET was robust.

It was the most significant predictor of PFS on univariate analysis (p=0.003) and retained its independent significance in multivariable analysis (p=0.023), whereas other factors were not significant (Table 5). 

 Subsequent subgroup analysis confirmed that iPET status superseded the IPS in predicting outcome, as no significant PFS difference was observed between IPS groups when stratified by iPET result (Figure 1). All patients were alive at the end of the study.

**Table 5:** The association between PFS with demographic variables, baseline clinical factors and iPET

**Unvariate analysis**	**R**	**Sig.**	**95% CI for Exp (B)**	
			**Lower**	**Upper**
Stage (early vs advanced)	0.250	0.012*	1.704	4.141
Lymphocyte x109/L(≥0.8 vs <0.8)	0.096	0.341	1.771	5.150
White blood cell (>15 vs ≤15)	0.003	0.973	0.667	1.962
Hemoglobin (≥10.5 vs <10.5 g/dL)	0.145	0.151	0.335	1.113
Albumin (≥ 4 vs <4 g/dL)	0.003	0.977	1.006	5.959
IPS 0-2 vs ≥ 3	0.346	0.045*	0.328	0.994
iPET (positive vs negative)	0.104	0.003*	1.242	1.998
**Multivariate analysis (Cox)**				
iPET	0.440	0.023*	1.012	1.286

(*): Statistical significance

## Discussion

 The main objective of this study was to evaluate the predictive utility of iPET in patients with cHL receiving ABVD chemotherapy, the most widely used regimen for this disease. We assessed the prognostic value of iPET using the DS, now the standard tool for interim response evaluation. Notably, iPET findings informed subsequent treatment per NCCN guidelines, allowing for real-time risk-adapted decisions and an accurate assessment of predictive value8. To our knowledge, this is the first iPET-guided study in a Southeast Asian cohort and among the few from non-Western, developing settings (14, 19, 20). 

 An effective early-response biomarker must differentiate patients who may benefit from de-escalated therapy from those requiring more intensive regimens. Numerous studies have shown that the IPS lacks adequate predictive power for such stratification (4, 21, 22). 

 Consistent with these findings, our study did not identify IPS as a significant prognostic factor for 3-year PFS. In both univariate and multivariate analyses (Table 5), IPS (low vs. high risk) was not associated with PFS, whereas iPET was an independent predictor (p<0.05).

 To further explore the prognostic performance of iPET, subgroup analyses were performed. Among iPET-positive patients, early-stage cases demonstrated unexpectedly favorable outcomes, with 3-year PFS of 85.7% in favorable and 87.5% in unfavorable subgroups. In contrast, advanced-stage iPET-positive patients had poorer outcomes, with 3-year PFS of 50.0% in high-risk IPS and 33.3% in low-risk IPS. Although not statistically significant due to small sample sizes, this trend supports iPET as a stronger prognostic determinant than traditional risk factors, particularly in advanced disease. Collectively, these results highlight the consistency of iPET across different risk strata and underscore its potential as a cornerstone of individualized, response-adapted management in Hodgkin lymphoma.

 These findings align with prior studies. Gallamini et al. demonstrated that iPET, rather than IPS, independently predicted outcome: iPET(–) patients had excellent survival, while iPET(+) patients had poor prognosis (13). 

 Similar results were reported by Hutchings et al. and Al-Ibraheem et al., who showed iPET outperformed clinical stage and extranodal status as a prognostic tool HL (14, 23). In our cohort, the 3-year PFS was significantly higher in iPET(–) versus iPET(+) patients (93.6% vs. 40.9%, p<0.0001), reinforcing iPET as a strong independent predictor of treatment failure (4, 10, 22, 23). These rates are comparable to those in the JID (95.0% vs. 13.0%) and Gallamini et al. (95.0% vs. 28.0%) studies (13).

 Traditionally, treatment response was assessed using criteria from the 1999 International Workshop or Gallamini’s earlier protocols (24, 25). However, the Deauville score offers improved accuracy for interpreting residual masses in both HL and NHL. In a comparative analysis, Metser et al. found DS had the highest sensitivity, specificity, and accuracy (0.93, 0.94, and 0.93, respectively) on lesion-level analysis (26). The Lugano 2014 guidelines further endorsed DS as the preferred method, recommending visual assessment alone for end-of-therapy evaluation, without requiring SUV quantification (27).

 In our study, the high 3-year overall survival (OS) rate of 100% reflects the effectiveness of ABVD-based therapy. The diagnostic performance of iPET was also high: sensitivity 72.3%, specificity 89.0%, negative predictive value (NPV) 93.0%, positive predictive value (PPV) 59%, and accuracy 86.0% (Table 4). 

 These values mirror those reported by Gallamini and others (10, 13, 14). In multivariate analysis, iPET remained a significant predictor of PFS. Al-Ibraheem et al. similarly reported 3-year PFS and OS of 91% and 95% in iPET(–) patients vs. 41% and 86% in iPET(+) cases (p<0.0001) (14). The high NPV (94%) and accuracy (91%) observed here are consistent with previously published data across different disease stages (10, 23, 28, 29).

 Recent studies have explored different iPET timing points. Al-Ibraheem et al. found comparable prognostic power between iPET-2 and iPET-4, both showing strong PFS differences between CMR and non-CMR groups (14). 

 Current NCCN and ESMO guidelines recommend iPET after two ABVD cycles (8, 9). 

 In our cohort, early response after two cycles was more common in early-stage patients (79.2%) than in advanced-stage patients (75%). 

 Yet, 15 early-stage patients had only partial or no metabolic response, highlighting a subgroup that may benefit from treatment intensification *(*Table.3*). *The high iPET(–) rate (78%) and excellent NPV support de-escalation trials using ABVD to minimize toxicity while maintaining efficacy (28, 30, 31). Gallamini et al.’s multicenter study of over 260 HL patients reported similar iPET response rates (17.3% iPET(+), 82.6% iPET(–)), validating the reproducibility of 5-point DS scoring when interpreted by experienced nuclear medicine specialists (13).

 In our study, treatment was tailored according to NCCN guidelines. iPET(–) patients continued ABVD or AVD, while most iPET(+) patients were escalated to BEACOPP, followed by response reassessment and, when needed, consolidation radiotherapy or salvage regimens. These results highlight the feasibility and prognostic relevance of NCCN-consistent, iPET-adapted strategies in resource-constrained settings like Vietnam.

 Several limitations should be acknowledged. First, heterogeneity in baseline staging due to inconsistent use of pre-treatment PET/CT may introduce unmeasured confounding. Subgroup analysis revealed no significant PFS differences between CT vs. PET/CT-staged patients (log-rank p>0.05), but limited PET/CT numbers reduce confidence. Second, as a single-center study in Vietnam, findings may not be generalizable. Nonetheless, the results support the applicability of response-adapted therapy in low-resource settings. Future prospective, multicenter studies with uniform staging and additional biomarkers (e.g., EBV) are needed to validate and expand these findings.

## Conclusion

 Interim PET/CT using Deauville criteria after two ABVD cycles demonstrated high prognostic accuracy across all risk categories, with the strongest performance observed in favorable early-stage and low-risk advanced-stage patients. It provides robust prognostic value and enables early response-adapted therapy in Hodgkin lymphoma. Our findings affirm the clinical applicability of NCCN-guided PET-based strategies in Southeast Asian settings, supporting their integration into context-specific treatment frameworks.

## Abbreviations

**[** ^18^ **F]FDG PET/CT: **[^18^F]-Fluoro Deoxy Glucose Positron Emission Tomography/ Computed Tomography

**SUV: **Standard Uptake Value

**ABVD: **Adriamycin, Bleomycin, Vinblastine sulfate, and Dacarbazine

**BEACOPP: **Bleomycin, Etoposide, Adriamycin, Cyclophosphamide, Vincristine, Procarbazine and Prednisone 

**CT: **Computed Tomography

**CMR: **Complete Metabolic Response 

**CHL: **Classical Hodgkin Lymphoma

**DS: **Deauville five-point Score

**EANM: **European Association of Nuclear Medicine

**ESMO:** European Society for Medical Oncology

**IPET: **Interim Positron Emission Tomography/ Computed Tomography

**IPS: **International Prognostic Score 

**NCCN: **National Comprehensive Cancer Network

**EFS: **Event-Free Survival 

**PFS: **Progression-Free Survival

**IFRT: **Involved-Field Radio Therapy 

**PMR: **Partial Metabolic Response 

**NMR: **No Metabolic Response 

**PMD: **Progressive Metabolic Disease 

**WHO: **World Health Organization
